# Random pharmacokinetic profiles of EC-MPS in children with autoimmune disease

**DOI:** 10.1186/1546-0096-8-1

**Published:** 2010-01-04

**Authors:** Guido Filler, Ajay Parkash Sharma, Deborah M Levy, Abeer Yasin

**Affiliations:** 1 Department of Pediatrics, University of Western Ontario, Ontario, Canada

## Abstract

**Background:**

Therapy with mycophenolate mofetil (MMF) has become a valuable therapeutic option in children with autoimmune disease. MMF prescription in children with autoimmune diseases differs from that in transplant recipients in terms of different dosing regimen, and concomitant administration of other immunosuppressive medications. Recently, another formulation of the same active compound, mycophenolic acid (MPA), has become available as enteric-coated Mycophenolate Sodium (EC-MPS). Dosing and pharmacokinetics of EC-MPS in pediatric autoimmune disease have never been studied.

**Methods:**

We therefore performed a pilot study on 6 patients, who were treated with EC-MPS. All patients underwent 1-2 full 10-point pharmacokinetic (PK) profiles over a 12-hour dosing interval. We compared the results with that of 22 similar patients on MMF therapy.

**Results:**

Median EC-MPS dose was 724 mg/m^2 ^(range 179-933 mg/m^2^). The MPA Area-Under-The-(Time-Concentration)-Curves (AUCs) on MMF and EC-MPS were comparable (54.4 mg × h/L on MMF and 44.0 mg × h/L on EC-MPS, n.s., Mann Whitney). After correcting for bioequivalence, the dose-normalized AUCs were also similar on both the formulations. However, PK profiles on EC-MPS were quite random, and time to maximum concentration varied from 30 minutes to 720 minutes. The concentration at six-hour correlated best with the AUC. This was different from a homogenous PK-profile on MPA.

**Conclusions:**

EC-MPS has a different PK profile from MMF. The data suggest that patients on EC-MPS must undergo a complete PK profile to assess adequate exposure. The 6-hour concentration provides an estimate of the exposure and should be targeted between 3-4 mg/L.

## Background

Mycophenolate mofetil (MMF) is an immunosuppressive drug that reversibly inhibits the inosin monophosphate dehydrogenase (IMPDH), thereby providing selective inhibition of the proliferation of B and T-cells as they require de-novo synthesis of purines [1]. MMF has become a valuable treatment option for adults and children with autoimmune diseases. A recent randomized controlled clinical trial suggests equal efficacy when compared to cyclophosphamide for the initial treatment of lupus nephritis [2]. There are few publications on the dosing of MMF. Based on pharmacokinetic studies in children with autoimmune disease, an initial dosing of MMF at 900 mg/m^2 ^in two divided doses is recommended [3]. This dose is lower than that recommended for pediatric renal transplant recipients, where the starting dose should be between 1200 and 2400 mg/m^2^, depending on the concomitant calcineurin inhibitor. The reasons for different dosing of MMF in pediatric rheumatology patients as compared to pediatric renal transplant patients is explained by the lack of a concomitant calcineurin inhibitor. There are drug-drug interactions between both calcineurin inhibitors and MMF that explain the variable dosing requirements [4].

The AUCs obtained with the pediatric MMF dose of 900 mg/m^2 ^in pediatric lupus patients compare favorably or slightly higher than those in adults on a dose of 1 g PO twice daily [5]. Therapeutic drug monitoring (TDM) of MMF therapy is recommended in patients with autoimmune disease and typically done by trough level monitoring because of high inter-individual variability and unpredictable MPA exposure with a fixed MMF dose while there is a concentration-effect relationship between the MPA trough level and immunological disease activity parameters [6].

Recently, a novel formulation of the active compound, mycophenolic acid (MPA), was introduced as enteric-coated mycophenolate sodium (EC-MPS). It was hoped that this compound reduced the frequent gastrointestinal side effects of MMF [7]. In the province of Ontario, the government no longer reimburses MMF for patients with autoimmune disease, thereby forcing physicians to prescribe EC-MPS instead. While the literature suggests that the pharmacokinetics are radically different in transplant recipients (reviewed in 5), studies on the pharmacokinetics of EC-MPS in children with autoimmune disease remain elusive. We compared the results of 5 patients with EC-MPS with historical PK profiles from 22 patients who received MMF therapy.

## Methods

### Patients

The previous study [3] that established the dosing of MMF in pediatric rheumatological patients was approved by the hospital Research Ethics Committee. As only six patients were on EC-MPS, and as pharmacokinetic monitoring of MMF and EC-MPS therapy is clinical routine in our unit, we did not seek ethics approval for the analysis of the PK profiles from these six patients. Of these six patients, three had Systemic Lupus Erythematosus (SLE), one had sarcoidosis and two had an autoimmune glomerulonephritis.

The control group with MMF therapy has been described earlier [3]. In addition to the 15 patients that were published, 7 additional patients with SLE were included. All underwent standard immunosuppressive treatment prior to MMF initiation. MMF (Cellcept^®^) was obtained from Roche Laboratories, Nutley, NJ, USA. Only 250 and 500 mg capsules were used. The patients on EC-MPS were treated with Myfortic^® ^obtained from Novartis Canada, Mississauga, ON, Canada, and 180 and 360 mg capsules were used.

### Methods

Standard laboratory test results were retrieved from the patient's files. All patients (on MMF or EC-MPS) underwent therapeutic MPA monitoring using trough levels with a commercially available automated EMIT assay [8]. After establishment of stable trough concentrations between 1 and 5 mg/L, all patients had at least one full pharmacokinetic (PK) profile. PK profiles were obtained after inserting an intravenous cannula and obtaining a baseline trough level (C0) in a fasting state usually early morning. The patients then took their usual dose of MMF or EC-MPS and immediately thereafter ate a standard meal. They had free access to non-dairy product drinks during the day and regular meals. EDTA whole blood samples (2 mL) were then taken for duplicate measurements of MPA concentration at 0.5, 1, 1.5, 2, 3, 4, 6, 8 and 12 hours, respectively for a 10 point 12 hour PK profile. The area under the curve was calculated according to the trapezoid rule.

### Statistics

All contiguous data were tested for normal distribution using the Kolmogorov Smirnov test. In case of normally distributed data, results were presented as mean ± standard deviation (SD), whereas non-normally distributed data were expressed as median and 25^th ^and 75^th ^percentile. Standard correlation analysis was performed using appropriate parametric or non-parametric approaches. Comparisons between groups were performed using t-test for normally distributed data and Mann-Whitney test otherwise. A p value of less than 0.05 was considered significant. All statistical analysis was performed using GraphPad Software for Science Version 4.01, San Diego, CA.

## Results

**Figure 1:**
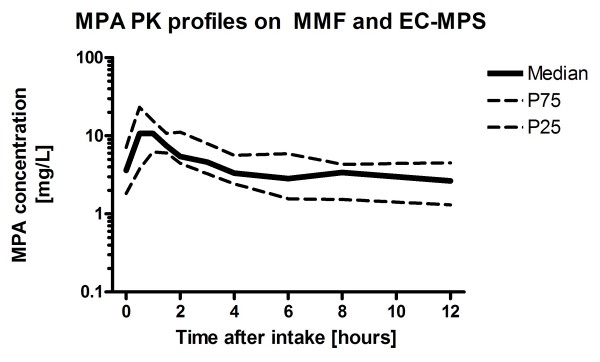
**The relationship between time after intake of MMF [hours] and the median MPA concentration as well as the 25th and 75th percentile**.

All variables under study were investigated for normality using the Kolmogorov Smirnov normality test and most variables were found to be not normally distributed. For consistency, all statistical measures of parameters were, therefore, reported as median, 25^th ^and 75^th ^percentile. The analysis of the PK profiles of the 22 patients with pediatric autoimmune disease is given in figure 1. Following oral administration, the drug is rapidly absorbed (median t_max _60 minutes, a second peak at 6 hours, figure 1).

By contrast, t_max _was later on EC-MPS with a median of 180.0 minutes (25^th ^percentile 60 minutes, 75^th ^percentile 240 minutes, range 30 to 720 minutes). Median C_max _was 20.1 mg/L (25^th ^percentile 10.3, 75^th ^percentile 45.1). The median pre-dose trough level C0 was 2.76 mg/L (25^th ^percentile 2.29, 75^th ^percentile 5.55). The median dose per m^2 ^body surface area had a median of 724.2 mg/m^2 ^(25^th ^percentile 660.2, 75^th ^percentile 859.2). MPA AUC on EC-MPS had a median of 44.0 mg × h/L (25^th ^percentile 42.0 mg × h/L, 75^th ^percentile 196 mg × h/L), not significantly different from the median AUC from the 22 patients on MMF who had a median AUC of 57.9 mg × h/L (unpaired t-test). The bioequivalent dose for 250 mg of MMF is 180 mg of EC-MPS. The median dose of 724.2 mg/m^2 ^would be equivalent to 1005.8 mg/m^2 ^of MMF which compared to a median of 893 mg/m^2 ^that the 22 patients on EC-MPS were given (not significant). The dose normalized MPA AUC had a median of 0.11 mg × m^2 ^× h/L × mg (25^th ^percentile 0.05, 75^th ^percentile 0.25 mg × m^2 ^× h/L × mg). The dose normalized MPA-AUC in the 22 patients on EC-MPS was 0.057 m^2 ^× h/L. After conversion of the of the EC-MPS dose to a bioequivalent dose of MMF, the dose normalized AUC no longer differed from that of the patients on MMF (bioequivalent EC-MPS AUC was 0.14 mg × m^2 ^× h/L × mg, p > 0.05, Mann Whitney test). This suggests that in pediatric patients with autoimmune disease the same conversion of the dose with 250 mg of MMF being equivalent to 180 mg of EC-MPS is applicable.

**Figure 2:**
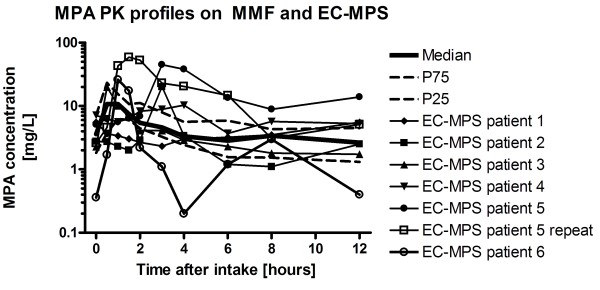
**Six individual pharmacokinetic PK profiles of 6 pediatric patients with autoimmune disease on EC-MPS, superimposed on figure 1**.

Patients on EC-MPS showed random PK profiles (figure 2). For the convenience of the reader, we superimposed the actual PK profiles over the percentiles of the MMF profiles.

We then studied whether the trough level could be used to assess the MPA exposure in patients on EC-MPS. Spearman correlation coefficients between AUC and the different C_n _trough level points revealed no correlation between the trough level. The best correlation was for the 6-hour concentration (Spearman r = 0.919). The median C_6 _level was 3.21 mg/L, the 25^th ^percentile for the C_6 _was 1.20 mg/L and the 75^th ^percentile was 13.5 mg/L. The results of the correlation studies are provided in table 1. AUC could not be estimated on the basis of the C_0_, C_1_, C_2_, or C_4 _concentrations, which are time points commonly used for limited sampling strategies. Based on the preliminary data from these 6 patients, the 6-hour concentration should be targeted between 3-4 mg/L.

**Table 1:** Correlation between exposure (12-hour AUC determined by full 10-point PK profile, calculated using the trapezoid rule) and individual time concentration points.

Parameter	C_0_	C_0.5_	C_1_	C_1.5_	C_2_	C_3_	C_4_	C_6_	C_8_	C_12_
Number of XY Pairs	7	7	7	7	7	7	7	7	7	7

Spearman r	0.3929	0.7500	0.2162	0.2162	0.8571	0.7857	0.8571	0.9190	0.6786	0.6786

P value (two-tailed)	0.3956	0.0663	0.6615	0.6615	0.0238	0.0480	0.0238	0.0067	0.1095	0.1095

P value summary	ns	ns	ns	ns	*	*	*	**	ns	ns

Exact or approximate P value?	Exact	Exact	Exact	Exact	Exact	Exact	Exact	Exact	Exact	Exact

Is the correlation significant? (alpha = 0.05)	No	No	No	No	Yes	Yes	Yes	Yes	No	No

Only C_6 _correlated with AUC.

## Discussion

The objective of the study was the characterization of the pharmacokinetics of EC-MPS in pediatric patients with autoimmune disease without a concomitant calcineurin inhibitor. As outlined in the introduction, this was necessary as special dosing is required and data from transplant patients cannot simply be applied to these patients. Secondary objectives were to determine whether the recommendations for bioequivalence of dosing can be applied to pediatric patients with autoimmune disease and whether trough level monitoring is feasible to study exposure in these children.

The study demonstrates that bioequivalent dosing of EC-MPS can indeed be implemented using the same conversion (180 mg of EC-MPS for 250 mg of MMF). AUC's achieved with a median dose of 712 mg/m^2 ^resulted in comparable MPA exposure. However, there was substantial inter-individual variability. Furthermore, t_max _was random and only the C_6 _concentration correlated significantly with the exposure.

A bioequivalent dose of 180 mg of EC-MPS being equivalent to 250 mg of MMF has been reported for transplant patients[9]. This has not been shown for pediatric rheumatology patients. This paper provides the first evidence that the same conversion can be applied with comparable AUCs. When correcting for the bioequivalence, the dose-normalized MPA AUCs are also similar. As such, the clinician may switch between both formulations should the gastrointestinal (GI) side effects prevent ongoing use of MMF. However, the jury remains out as to whether conversion to EC-MPS improves the GI side effects [10]. We are unaware of any study in SLE patients that compares GI side effects of both formulations. The GI tolerability of MMF therapy appears less of a problem than in transplant patients, possibly because of the use of concomitant calcineurin inhibitors [11].

Importantly, there was substantial inter-patient variability. There was a wide range of the dose-normalized AUC (maximum 5.6 higher than minimum), confirming data on liver transplant patients that suggest wide inter-patient variability [12]. These data stress the need for TDM in pediatric rheumatology patients as has also been shown for adult patients [6]. As with MMF, we recommend that at least one full PK profile be obtained on stable dosing in these patients [3]. Unfortunately, trough level monitoring, which forms the mostly widely used TDM strategy, is not feasible in these patients. There is absolutely no correlation at all between AUC and C_0 _or C_12_. It is well established that trough level monitoring of MPA is insufficient in transplant recipients [13]. Limited sampling strategies are used instead, most commonly involving C_0_, C_1_, C_2 _and C_4 _[14]. Based on the preliminary results in this study, these strategies will fail. Only the C_6 _concentration correlates significantly with the trough level. Our study provides insufficient numbers to establish a limited sampling model based on the C_6 _concentration. The authors encourage multi-center studies on pediatric rheumatology patients on EC-MPS to establish such strategies. Until these become available, a full PK profile should be entertained to determine that patients have adequate exposure.

The question about what exactly comprises adequate exposure remains to be determined. Recent evidence from transplant studies suggests that an AUC greater than 30 mg × h/L is required to prevent rejection [15]. However, no upper therapeutic window could be established. Generally, SLE patients are aimed at a higher AUC [6], and the therapeutic window has not yet been formally confirmed in randomized controlled clinical trials. It appears that the EC-MPS dose of 712 mg/m^2 ^may provide adequate exposure with a target AUC of 60 mg × h/L. None of the patients relapsed on the respective doses with their measured AUCs, but the follow-up is short (367 days, range 106-612 days, data not shown in results) and the numbers are insufficient to draw any conclusions.

Our study is limited by the small number of patients studied. Nonetheless, the observation of the random t_max _and the very substantial inter-patient variability clearly warrants the need for TDM in these patients. The limited sampling strategies typically employed for MMF therapy are inadequate, thus further studies are needed to assess the feasibility of the 6-hour concentration. The authors stress the need for TDM in these patients using full PK profiles.

## Conclusion

A full pharmacokinetic profile is required to assess the MPA exposure when treating patients with EC-MPS. The 6-hour concentration can provide a rough estimate of this exposure. Based on the preliminary data from only 6 patients, the 6-hour concentration should be targeted between 3-4 mg/L.

## Abbreviations

AUC: Area under the curve; C0: pre-dose trough level; C_max_: maximum concentration; C [n]: level drawn at [n] hours after oral intake - example; C2: concentrations at 2 hours; EC-MPS: Enteric coated mycophenolate sodium; EMIT: enzyme-mediated immunoassay technique; IMPDH: Inosine Monophosphate Dehydrogenase; MMF: Mycophenolate mofetil; MPA: Mycophenolic acid; r: Spearman rank r; SLE: systemic lupus erythematosus; t_max_: time to maximum concentration after oral intake; TDM: Therapeutic drug monitoring.

## Competing interests

The authors declare that they have no competing interests.

## Authors' contributions

GF designed the study, collated the data, performed the analysis, and drafted the manuscript. AY performed the statistical analysis and helped to draft the manuscript. DL in the design of the study and carefully edited the manuscript. AS participated in the design of the study and coordination and helped to draft the manuscript. All authors read and approved the final manuscript.
